# EXAMINING THE RELATIONSHIP BETWEEN COGNITIVE FUNCTIONING AND SUBJECTIVE WELL-BEING ACROSS AGE

**DOI:** 10.1093/geroni/igz038.2408

**Published:** 2019-11-08

**Authors:** Francesca Falzarano, Jillian Minahan, Neshat Yazdani, Karen L Siedlecki, Timothy Salthouse

**Affiliations:** 1 Fordham University, Bronx, New York, United States; 2 University of Virginia, Charlottesville, Virginia, United States

## Abstract

Higher levels of subjective well-being (SWB) are associated with myriad of positive outcomes, including better physical health. Several variables have been shown to predict SWB, including cognitive functioning. The relationship between aspects of SWB (positive affect, negative affect, and life satisfaction) and cognition were examined in participants (N = 5, 125) between the ages of 18- 99 years from the Virginia Cognitive Aging Project (VCAP). Participants completed a battery of cognitive tasks, including tests of verbal episodic memory, processing speed, reasoning, spatial visualization, and vocabulary. Cross-sectional analyses were conducted using structural equation modeling, using full information maximum likelihood estimation. In the models, the five latent cognitive constructs simultaneously predicted each of the SWB outcome variables separately. Age, education, gender, and self-rated health were included as covariates. Results show that reasoning was a significant unique predictor of negative affect (-.30), vocabulary was a significant unique predictor of positive affect (-.21), and spatial visualization was a significant unique predictor of life satisfaction (.21). Age moderation was examined by dividing the sample into three age groups (younger, middle-aged, and older). There was some evidence of age moderation. Namely, spatial visualization was a significant unique predictor of life satisfaction in the younger sample only. Reasoning and processing speed predicted negative affect in the younger group, whereas only reasoning predicted negative affect in the older group. In conclusion, in a large community-based sample spanning adulthood, there is evidence that cognition predicts aspects of SWB but there is variation across SWB outcome variables, and across age.

